# Application of Bayesian analysis to the doubly labelled water method for total energy expenditure in humans

**DOI:** 10.1002/rcm.8013

**Published:** 2017-11-23

**Authors:** Yue Ruan, Les C.J. Bluck, James Smith, Adrian Mander, Priya Singh, Michelle Venables

**Affiliations:** ^1^ University of Cambridge Metabolic Research Laboratories, Wellcome Trust‐MRC Institute of Metabolic Science and Department of Paediatrics University of Cambridge Cambridge CB2 0QQ UK; ^2^ MRC Elsie Widdowson Laboratory (formerly MRC Human Nutrition Research) Fulbourn Road Cambridge CB1 9NL UK; ^3^ School of Food Science and Nutrition, Faculty of Mathematics and Physical Sciences University of Leeds Leeds LS2 9JT UK; ^4^ MRC Biostatistics Unit Cambridge Institute of Public Health Forvie Site Robinson Way, Cambridge Biomedical Campus Cambridge CB2 0SR UK

## Abstract

**Rationale:**

The doubly labelled water (DLW) method is the reference method for the estimation of free‐living total energy expenditure (TEE). In this method, where both ^2^H and ^18^O are employed, different approaches have been adopted to deal with the non‐conformity observed regarding the distribution space for the labels being non‐coincident with total body water. However, the method adopted can have a significant effect on the estimated TEE.

**Methods:**

We proposed a Bayesian reasoning approach to modify an assumed prior distribution for the space ratio using experimental data to derive the TEE. A Bayesian hierarchical approach was also investigated. The dataset was obtained from 59 adults (37 women) who underwent a DLW experiment during which the ^2^H and ^18^O enrichments were measured using isotope ratio mass spectrometry (IRMS).

**Results:**

TEE was estimated at 9925 (9106‐11236) [median and interquartile range], 9646 (9167–10540), and 9,638 (9220–10340) kJ·day^−1^ for women and at 13961 (12851–15347), 13353 (12651–15088) and 13211 (12653–14238) kJ·day^−1^ for men, using normalized non‐Bayesian, independent Bayesian and hierarchical Bayesian approaches, respectively. A comparison of hierarchical Bayesian with normalized non‐Bayesian methods indicated a marked difference in behaviour between genders. The median difference was −287 kJ·day^−1^ for women, and −750 kJ·day^−1^ for men. In men there is an appreciable compression of the TEE distribution obtained from the hierarchical model compared with the normalized non‐Bayesian methods (range of TEE 11234–15431 kJ·day^−1^ vs 10786–18221 kJ·day^−1^). An analogous, yet smaller, compression is seen in women (7081–12287 kJ·day^−1^ vs 6989–13775 kJ·day^−1^).

**Conclusions:**

The Bayesian analysis is an appealing method to estimate TEE during DLW experiments. The principal advantages over those obtained using the classical least‐squares method is the generation of potentially more useful estimates of TEE, and improved handling of outliers and missing data scenarios, particularly if a hierarchical model is used.

## 1 INTRODUCTION

The doubly labelled water (DLW) technique of indirect calorimetry for the estimation of total energy expenditure (TEE) was originally suggested by Lifson et al^1^ and applied to use in humans some time later.^2^, ^3^ It is now a well‐established method and considered a gold‐standard for the measurement of TEE under free‐living conditions.^4^

The main assumptions of the DLW method originally provided by Lifson and McClintock^5^ have been more recently summarized and scrutinized by Coward and Cole,^6^ who concluded that, whilst none of the six basic assumptions were true, at least the imperfections were manageable.

Although at least three important works describing the principles and practices of the DLW method, striving to promote universal consistency, have been produced, there is still some non‐uniformity in the calculations adopted by workers at different laboratories. This is particularly the case for corrections for fractionation (Assumptions 1 to 3 for space ratios and Assumption 5 are discussed by Coward and Cole^6^). The major difficulty in dealing with fractionation is the estimation of the proportion of water that undergoes phase change (from liquid to vapour) before being lost from the body. This is to some extent dependent on the environment of subjects and their physical activity and this needs to be considered within a given experimental paradigm.

The approach adopted for the space ratio, however, is less open to modulation by the experimental environment. When body water is estimated from an isotope dilution experiment, the value obtained is an over‐estimate by a factor of approximately 4% if ^2^H is used or about 1% when ^18^O is employed. For ^2^H, this is attributed to the exchange with labile hydrogen atoms, principally from proteins and lipids.^7^ The ^18^O pool size exceeds that of the body water pool, not only because of the exchange with dissolved CO_2_ and bicarbonate,^8^ which is fundamental to the principle of the DLW method, but also because of exchange with bone mineral and other deep pools. The practical consequence of this is that neither the accessible ^2^H nor the ^18^O volumes of distribution (pools) are coincident with the total body water, and furthermore there is a measurable difference between the apparent volumes into which the two isotopes are distributed.

These issues are discussed by Coward^9^ alongside the recommendation of Schoeller et al.,^10^ that a fixed ratio of 1.03 (later revised to 1.034^11^ and further to 1.036^11^) between the ^2^H space (*N*_H_) and the ^18^O space (*N*_O_) be adopted. It is suggested that the experimental space ratio should be used as a screen for the quality of the DLW data, with values lying outside the range of between 1.015 and 1.060 indicating potential dosing error or analytical error. If the spaces have been deduced from back‐extrapolation of a linear fit to the semi‐logarithmically transformed curves of disappearance, the experimental values should be used directly. On the other hand, if the spaces are deduced from enrichments in the first few hours post‐dose (the plateau method) with flux derived separately, the suggestion of Schoeller et al. should be adopted and the pool sizes combined to achieve the desired ratio. In practice, this is achieved by weighting the experimentally obtained values according to:

(1) $$N_{H}\left(\text{corr}\right)=\frac{1}{2}\left(N_{H}\left(\mathrm{obs}\right)+1.03N_{O}\left(\mathrm{obs}\right)\right)$$

(2) $$N_{O}\left(\text{corr}\right)=\frac{1}{2}\left(\frac{N_{H}\left(\mathrm{obs}\right)}{1.03}+N_{O}\left(\mathrm{obs}\right)\right)$$

Speakman^12^ discusses comprehensively the correctness of this approach, with the tentative conclusion that in humans the fixed ratio approach should be used, but with a modified coefficient derived from the mean experimental ratio found for the given sub‐population under study.

The International Atomic Energy Agency (IAEA)^13^ advocates universal adoption of the equations:

(3) $$N_{H}\left(\text{corr}\right)=1.041\times \frac{1}{2}\left(\frac{N_{H}\left(\mathrm{obs}\right)}{1.041}+\frac{N_{O}\left(\mathrm{obs}\right)}{1.007}\right)$$

(4) $$N_{O}\left(\text{corr}\right)=1.007\times \frac{1}{2}\left(\frac{N_{H}\left(\mathrm{obs}\right)}{1.041}+\frac{N_{O}\left(\mathrm{obs}\right)}{1.007}\right)$$

which fixes the space ratio at a value of 1.034, as suggested by Racette et al.^14^

The consequences of the decision to normalize the space ratios are not trivial and affect the estimation of the TEE. Clearly, decisions made in deriving the TEE from the experimental isotope enrichments are important, and yet are frequently undocumented in publications.

In this work, we develop the estimation of TEE further. Previously, we demonstrated the use of Bayesian methods in modelling gastric emptying,^15^, ^16^ and their use in other tracer methods have been assessed.^17^ For doubly labelled water, Bayesian methods are very attractive as there is a considerable amount of prior knowledge for any experiment. Apart from the space ratio, which can be assigned a distribution, approximate values for water turnover and lean body mass can be predicted *a priori* from anthropometric parameters.

The aim of the present study was therefore to develop and implement a more informative Bayesian model for the calculation of TEE. A convenient implementation of Bayesian statistics employing Monte Carlo Markov Chain algorithms is provided by the WinBUGS package^18^ used in this work.

## 2 EXPERIMENTAL

### 2.1 Data and instrumentation

The data used for this study was taken from the Adults aged 19–64 years NDNS survey of 1999–2000.^19^ Isotope ratios were measured using isotope ratio mass spectrometry (AP2003 mass spectrometer; Analytical Precision, Manchester, UK, with an analytical precision better than ±0.12 ‰, for *δ* ^18^O values and Aqua‐SIRA mass spectrometer, VG Isogas, Middlewich, UK, with an analytical precision of ±1.5 ‰, for *δ* ^2^H values) using equilibration methods for oxygen^20^ and reduction over uranium for hydrogen.^21^ All data are expressed relative to the international standard Vienna Standard Mean Ocean Water (vSMOW).

### 2.2 Non‐Bayesian equations for $R_{{\mathrm{CO}}_{2}}$ determination

Non‐normalized $R_{{\mathrm{CO}}_{2}}$ has been calculated using the equation of Coward:^22^

$$R_{CO_{2}}=\frac{k_{O}N_{O}-k_{H}N_{H}-27.3\left(f_{2}-f_{1}\right)}{2f_{3}+1.1\left(f_{2}-f_{1}\right)}$$

where *k* and *N* refer to the rate constant and pool size, respectively, with subscripts to indicate the isotope.

However, normalized $R_{{\mathrm{CO}}_{2}}$ has been calculated using the equation of Schoeller et al:^10^

$$R_{CO_{2}}=\frac{k_{O}N_{O}-k_{H}N_{H}}{2f_{3}+2.1\left(f_{2}-f_{1}\right)}$$

where *k* refers to the rate constant, *N* refers to the normalized pool size which fixes the space ratio at 1.03, and the subscripts indicate the isotope.

The fractionation factors *f*_1_, *f*_2_ and *f*_3_ are given as 0.941, 0.991 and 1.037, respectively.

### 2.3 Re‐parameterization of the DLW equations

For ease of model specification in the Bayesian environment, the first step is to re‐cast the DLW equations such that the observed mass spectrometric enrichments are expressed in terms of the parameters of physiological relevance. Since it is assumed that for all stable isotopes employed, including ^2^H and ^18^O, elimination from the body follows first‐order kinetics, the expression for the MS‐derived enrichment of each isotope at time *t* is of the form:

(5) $$\delta \left(t\right)=\frac{\mathrm{DT}\left(\delta_{\mathrm{dd}}-\delta_{T}\right)}{18.02\mathrm{Nd}}\exp\left\{-\mathrm{kt}\right\}+\delta_{b}$$

In deriving Equation (5), it is assumed that the usual method of combining the *δ*‐values (‰) of the sample of body water, *δ*(*t*) with the basal (pre‐dose) value *δ*_b_, and that of a diluted sample of the dose, *δ*_dd_, made by adding *d* grams to a quantity *T*, of naturally abundant water of known enrichment *δ*_T_ is used. The actual dose administered to the subject is *D* grams, the isotope space is denoted *N* (mol), and the fractional rate constant of elimination labelled as *k* (day^−1^).

The DLW technique combines the data from the two isotopes ^2^H and ^18^O to derive essentially four parameters: the CO_2_ product (R):*

(6) $$R_{{\mathrm{CO}}_{2}}=\alpha_{1}\left(k_{O}N_{O}-k_{H}N_{H}\right)+\alpha_{2}$$

the space ratio, *S*

(7) $$S=\frac{N_{H}}{N_{O}}$$

The water turnover, *R*_W_

(8) $$R_{W}=\beta_{1}k_{H}N_{H}+\left(1-\beta_{1}\right)k_{O}N_{O}+\beta_{2}$$

and the fraction of body fat *F*

(9) $$F=1-\frac{\gamma_{1}}{W}N_{H}-\frac{\gamma_{2}}{W}N_{O}$$

where the subject's body weight is *W*, and *α*_1_, *α*_2_, *β*_1_, *β*_2_, *γ*_1_ and *γ*_2_ are constants that depend upon the fractionation model employed. The parameter *F* is not necessary to calculate the TEE in this model, but its inclusion allows a further useful outcome from the ^2^H dataset.

The Total Energy Expenditure (TEE) is derived from $R_{{\mathrm{CO}}_{2}}$ as proposed by the modified Weir equation:^23^

(10) $$\mathrm{TEE}=22.4\times \left(\frac{15.48}{\mathrm{RQ}}+5.55\right)R_{{\mathrm{CO}}_{2}}$$

In this instance, we assumed a common respiratory quotient, *RQ* = 0.85 for all subjects, and therefore, TEE bears a constant ratio to $R_{{\mathrm{CO}}_{2}}$ with a constant of proportionality equal to 532.

Using simple algebra (see section S1, supporting information):

(11) $$N_{H}=\frac{\mathrm{WS}\left(1-F\right)}{\gamma_{1}S+\gamma_{2}}$$

(12) $$k_{H}=\frac{\left(\gamma_{1}S+\gamma_{2}\right)}{W\left(1-F\right)}\frac{\left[\alpha_{1}\left(R_{w}-\beta_{2}\right)-\left(1-\beta_{1}\right)\left(R_{{\mathrm{CO}}_{2}}-\alpha_{2}\right)\right]}{\alpha_{1}S}$$

(13) $$N_{O}=\frac{W\left(1-F\right)}{\gamma_{1}S+\gamma_{2}}$$

(14) $$k_{O}=\frac{\left(\gamma_{1}S+\gamma_{2}\right)}{W\left(1-F\right)}\frac{\left[\alpha_{1}\left(R_{w}-\beta_{2}\right)+\beta_{1}\left(R_{{\mathrm{CO}}_{2}}-\alpha_{2}\right)\right]}{\alpha_{1}}$$

The derivation of Equations (11)–(14) allows values to be sampled from prior distributions of the physiologically relevant parameters to make predictions of the observed kinetics. This therefore allows the generation of Bayesian estimates of the model parameters that derive TEE.

### 2.4 Choice of priors

In Bayesian analysis, the choice of priors for the physiological parameters of interest is of paramount importance; for the DLW model described here, vague (non‐informative) priors have been adopted for the parameters $R_{{\mathrm{CO}}_{2}},$ *R*_w_ and *F*,

$$\begin{array}{ll} 0<R_{{\mathrm{CO}}_{2}}<100\mathrm{mol}/\mathrm{day} & R_{{\mathrm{CO}}_{2}}∼\text{dunif}\left(0,100\right)\\ 0<R_{W}<1000\mathrm{mol}/\mathrm{day} & R_{W}∼\text{dunif}\left(0,1000\right)\\ 0<F<1 & F∼\text{dunif}\left(0,1\right) \end{array}$$

These priors allow the iterations to adopt values for these parameters that are almost entirely data driven. Note that a slightly different approach is used for the space ratio *S*. According to our prior knowledge, we suggested that *S* had a prior distribution that was normal, with a mean of 1.035 and with a standard deviation of 0.01 (precision = 10000), giving 99% confidence limits of 1.005 and 1.065. Therefore:

$$S∼\text{dnorm}\left(1.035,10000\right).$$

All the measured *δ* values were assumed to be normally distributed about the experimental value, with a prior standard deviation of 2 ‰ for ^2^H and 0.5 ‰ for ^18^O.

For the additional hierarchical analysis (see section S2, supporting information), hyper‐parameters (population parameters) adopt these distributions with the individual parameters drawn from them and associated with normal distributions:

$$\begin{array}{ll} R_{{\mathrm{CO}}_{2}}\left[i\right]∼\text{dnorm}\left(R_{{\mathrm{CO}}_{2}},{\mathrm{tau}}_{R_{{\mathrm{CO}}_{2}}}\right) & \;{\mathrm{tau}}_{R_{{\mathrm{CO}}_{2}}}∼\text{dgamma}\left(0.01,0.01\right)\\ R_{w}\left[i\right]∼\text{dnorm}\left(R_{w},{\mathrm{tau}}_{R_{w}}\right) & {\mathrm{tau}}_{R_{w}}∼\text{dgamma}\left(0.01,0.01\right)\\ F\left[i\right]∼\text{dnorm}\left(F,{\mathrm{tau}}_{F}\right) & {\mathrm{tau}}_{F}∼\text{dgamma}\left(0.01,0.01\right)\\ S\left[i\right]∼\text{dnorm}\left(S,{\mathrm{tau}}_{S}\right) & {\mathrm{tau}}_{S}∼\text{dunif}\left(1,100000\right) \end{array}$$

The between‐subject variance for the space ratio again reflects the richness of prior information for this variable.

### 2.5 Implementation in WinBUGS and description of datasets

For an initial investigation of the performance of the Bayesian methods, the three subjects used as examples given by Cole and Coward in Prentice^9^ were used as the error structures of various models used to interpret these data and are extensively discussed. Unfortunately, no anthropometric parameters are given for these examples, so an arbitrary weight of 70 kg was assigned to each subject. A second investigation used the same model as the first, but took as the dataset a cohort of 59 adults aged between 19 and 64 years, including 37 women and 22 men.

WinBUGS was installed on a 32‐bit standard laptop (Latitude E5410, Dell Computers Ltd, Bracknell, UK) running Windows 7 (Microsoft Corp., Redmond, WA, USA). For this application, 50,000 iterations were employed in the Markov Chain, with the first 4000 being discarded since they were regarded as 'burn in'. The code was written such that data for the 59 subjects in the large dataset were analyzed in a single programme run, which took 791 seconds. Whilst it was possible to analyze all 59 adults in a hierarchical fashion, we chose to perform a separate hierarchical analyses for men and women as the CO_2_ production rates and body compositions were expected to be drawn from different global distributions. Hierarchical analysis took 257 seconds for the 37 women and only 152 seconds for the 22 men.

## 3 RESULTS

Table 1 compares the results of the Bayesian analysis with those obtained by least‐squares analysis. In preparing this table, the results given in Table 11.2 of Prentice^9^ have been used to estimate *TEE* using the equations given in the first section. For all three subjects the Bayesian analysis returned estimates of TEE with uncertainty in the range from 4 to 8%. This is comparable with the estimated error obtained from logarithmic least‐squares by the method of Cole and Coward,^24^ which we calculate as 3.7%, 3.5% and 8.5% for subjects 1, 2 and 3, respectively.

**Table 1:** Results of parameter estimations obtained for three subjects using different models for deriving pool sizes and rate constants

Subject 1	Logarithmic		Poisson		Exponential		WinBUGS		
	Natural	Normalized	Natural	Normalized	Natural	Normalized	Mean	σ	CV
***N***_**H**_	2528	2531	2524	2525	2519	2520	2526	9	0.4%
***N***_**O**_	2451	2448	2444	2443	2438	2437	2446	10	0.4%
***k***_**H**_	0.0828	0.0828	0.0831	0.0831	0.0834	0.0834	0.0838	0.0004	0.5%
***k***_**O**_	0.1078	0.1078	0.1082	0.1082	0.1088	0.1088	0.1098	0.0006	0.6%
***S***	1.032	1.034	1.033	1.034	1.034	1.034	1.033	0.005	0.5%
$R_{{\mathrm{CO}}_{2}}$ **(mol.day^−1^)**	25.05	24.81	25.08	24.95	25.23	25.21	25.99	1.15	4.4%
***TEE*** **(kJ.day^−1^)**	13331	13202	13349	13278	13429	13417	13830	611	4.4%

Subject 2	Logarithmic		Poisson		Exponential		WinBUGS		
	Natural	Normalized	Natural	Normalized	Natural	Normalized	Mean	σ	CV
***N***_**H**_	2711	2720	2712	2711	2712	2701	2714	14	0.5%
***N***_**O**_	2640	2631	2620	2622	2603	2613	2622	12	0.4%
***k***_**H**_	0.1149	0.1149	0.1148	0.1148	0.1148	0.1148	0.1149	0.0008	0.7%
***k***_**O**_	0.1349	0.1349	0.1363	0.1363	0.1382	0.1382	0.1412	0.0009	0.7%
***S***	1.027	1.034	1.035	1.034	1.042	1.034	1.035	0.006	0.6%
$R_{{\mathrm{CO}}_{2}}$ **(mol.day^−1^)**	20.38	19.34	20.89	21.10	22.11	23.34	26.72	1.96	7.3%
***TEE*** **(kJ.day^−1^)**	10846	10294	11114	11230	11768	12423	14220	1043	7.3%

Subject 3	Logarithmic		Poisson		Exponential		WinBUGS		
	Natural	Normalized	Natural	Normalized	Natural	Normalized	Mean	σ	CV
***N***_**H**_	1856	1811	1846	1794	1839	1773	1822	12	0.7%
***N***_**O**_	1708	1752	1686	1735	1651	1715	1720	13	0.8%
***k***_**H**_	0.0892	0.0892	0.0899	0.0899	0.0903	0.0903	0.0904	0.0008	0.9%
***k***_**O**_	0.1342	0.1342	0.1358	0.1358	0.1398	0.1398	0.1482	0.0022	1.5%
***S***	1.087	1.034	1.095	1.034	1.114	1.034	1.059	0.008	0.7%
$R_{{\mathrm{CO}}_{2}}$ **(mol.day^−1^)**	29.20	33.87	28.98	34.33	29.78	36.77	41.66	2.11	5.1%
***TEE*** **(kJ.day^−1^)**	15542	18024	15425	18271	15845	19571	22170	1122	5.1%

When isotope data for a cohort of 37 women and 22 men was analyzed using classical least‐squares methods, the space ratio was found to vary between 1.010 and 1.069, with the majority falling in the ‘acceptable range’ judged by the criterion of Prentice.^9^ Using Coward's analysis, the mean estimates of TEE obtained using non‐normalized (natural) spaces were 9741 (women) and 13951 (men) kJ·day^−1^. When the space ratio was normalized, however, the corresponding estimates of TEE became 10080 (women) and 14573 (men) kJ·day^−1^. Normalization, therefore, increased the population estimate of TEE by approximately 4%.

**Figure 1:**
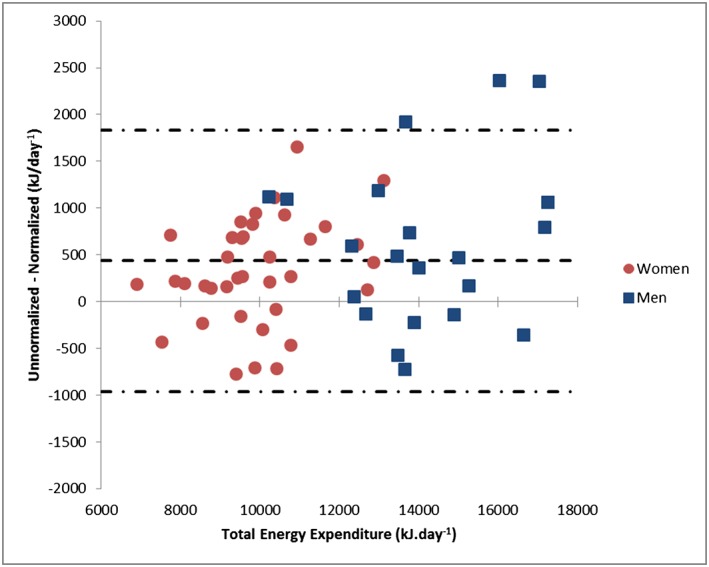
Bland‐Altman plot of Total Energy Expenditure obtained using non‐normalized and normalized body water spaces in 37 adult women and 22 adult men [Color figure can be viewed at wileyonlinelibrary.com]

**Figure 2:**
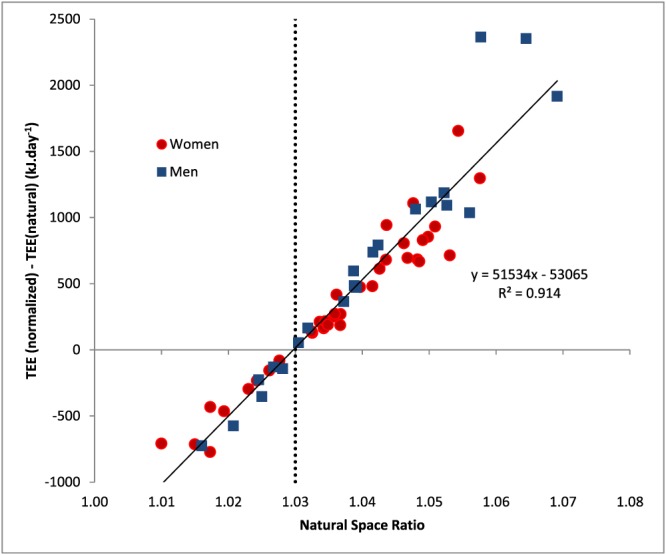
The effect of normalization of the space ratio on estimated TEE [Color figure can be viewed at wileyonlinelibrary.com]

The effects of normalization on the individual estimates of TEE are shown in Figure 1. From Figure 2 it is apparent that normalizing the space ratio decreases the estimated TEE if the natural space ratio is less than the target normalization, whilst the TEE is increased if the space ratio is more than the target normalization. Furthermore, this effect is highly linear.

**Figure 3:**
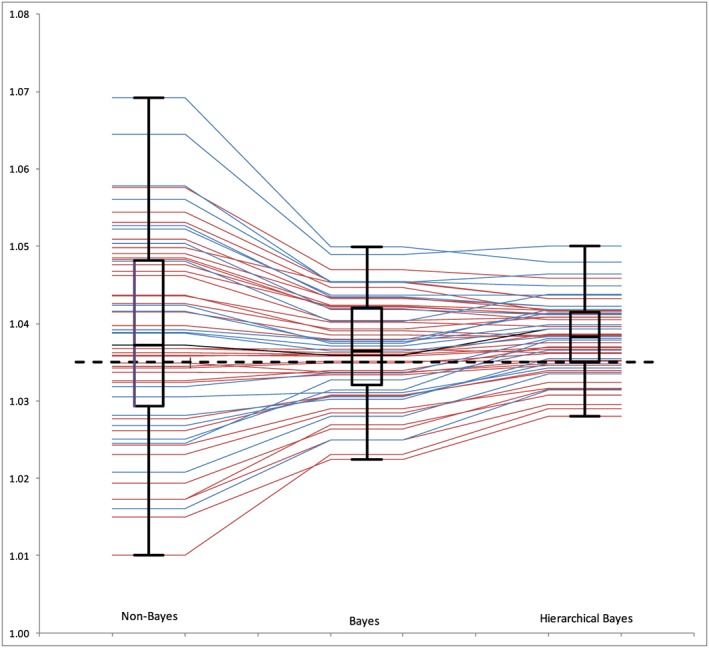
The space ratios for the pooled subjects obtained from non‐normalized non‐Bayesian, independent Bayesian, and hierarchical Bayesian analyses [Color figure can be viewed at wileyonlinelibrary.com]

**Figure 4:**
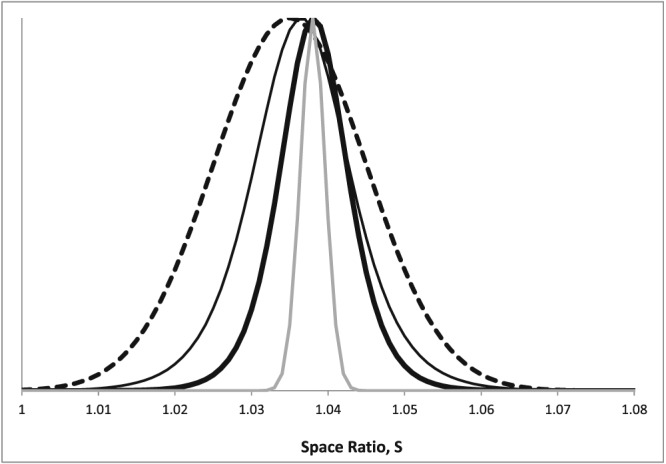
The prior (dotted line) and posterior distributions for the independent Bayesian analysis (thin line) and hierarchical Bayesian analysis (heavy line). Also shown is the distribution found from the hierarchical analysis for the hyperparameter *S*_*g*_

**Figure 5:**
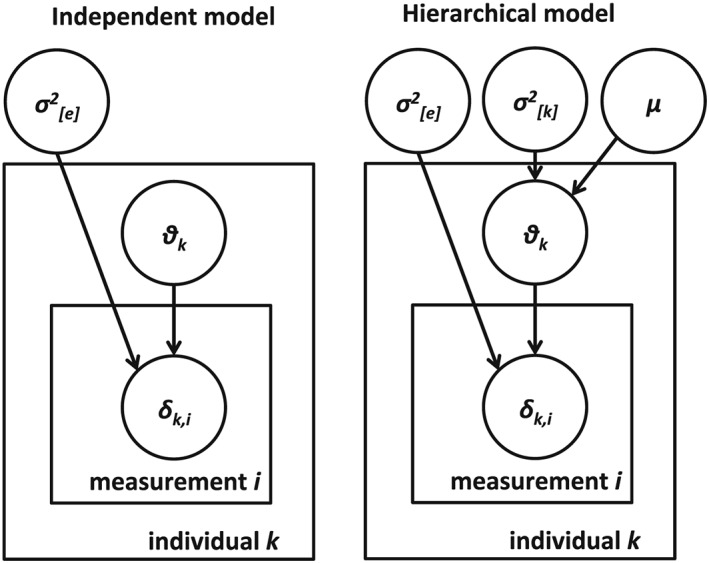
*δ_k,i_* is the oxygen/hydrogen isotope measurement for individual *k* at time *i*; *σ* ^2^_[*e*]_ represents the isotope measurement error; *θ_k_ =* [ $R_{{\mathrm{CO}}_{2}}$_*,k*_, *S_k_*, *R_W,k_*, *F_k_*], representing model parameter vector for subject *k*; and *μ* and *σ* ^2^_[*k*]_ are population mean and population variability representing the population distribution from which the individual parameters were drawn

**Figure 6:**
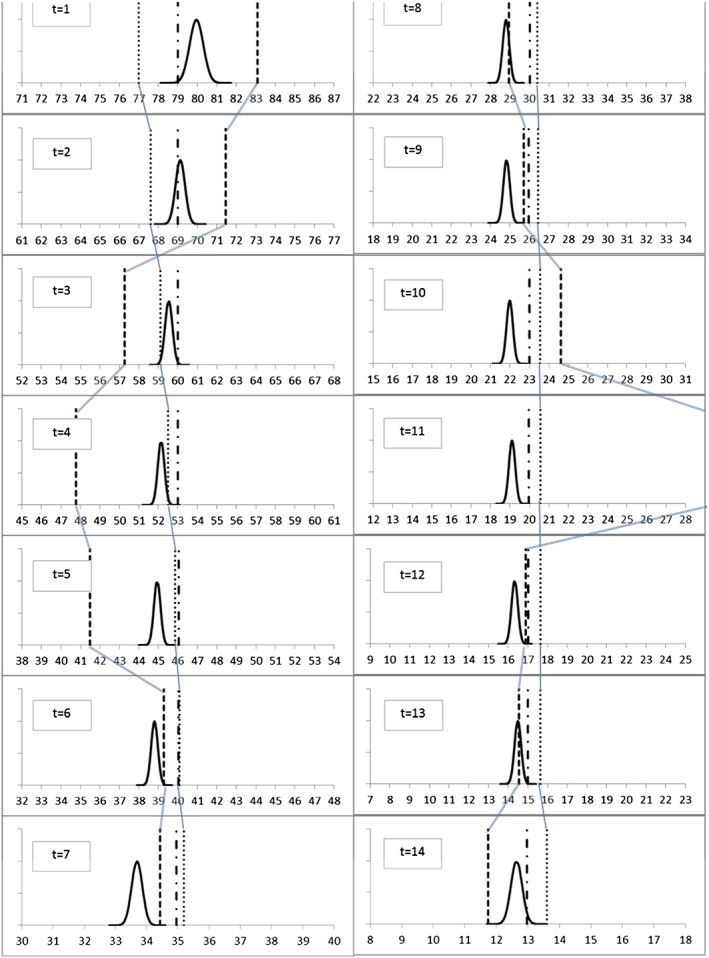
Observed and estimated ^18^O isotopic enrichments drawn from the data supplied from subject 3. Each graph represents a different *post‐dose* timepoint, and is drawn on the delta‐scale, centred about the predicted value from the unnormalized (exponential) model (shown as the chained line). The predictions from logarithmic transformation are indicated by the dotted lines and the experimental data shown by the dashed lines. The posterior distributions obtained from the Bayesian analysis are drawn as solid curves [Color figure can be viewed at wileyonlinelibrary.com]

When the independent Bayesian method was applied, as expected, the continuous distribution of individual median estimates of space ratios decreased (Table 2). A further reduction in the width of the distribution is achieved by specifying a hierarchical model (Figure 3). Since the distribution of space ratios was not found to be gender‐specific (Figure 2), the results for the women and men have been combined in Figure 3. The posterior distributions are drawn in Figure 4, along with the prior for comparison. Under the hierarchical analysis, a distribution is also obtained for the hyperparameter, *S*_g_, which was found to have a mean value of 1.0382, and a standard deviation of 0.0016. Plate model descriptions are found in Figure 5. Figure 6 shows observed and estimated ^18^O isotopic enrichments drawn from the data supplied from subject 3.

**Table 2:** The physiological parameters (median and range) obtained from the isotope data under the various methods of analysis

		Non‐normalized non‐Bayesian	Normalized non‐Bayesian	Independent Bayesian	Hierarchical Bayesian
***S***	**Women** a	1.037 (1.010–1.058)	1.035	1.036 (1.022–1.047)	1.037 (1.028–1.043)
	**Men** b	1.041 (1.016–1.069)	1.035	1.037 (1.025–1.050)	1.040 (1.031–1.050)
	**Combined** c	1.037 (1.010–1.069)	1.035	1.036 (1.022–1.050)	1.038 (1.027–1.049)
***F*** **(%)**	**Women** a	37.6 (23.2–51.8)	37.6 (23.2–51.8)	37.7 (23.2–51.6)	37.7 (23.2–51.6)
	**Men** b	27.4 (11.1–40.0)	27.4 (11.1–40.0)	27.4 (11.0–40.0)	27.4 (11.2–40.0
$R_{{\mathrm{CO}}_{2}}$ **(mol.day^−1^)**	**Women** a	18.1 (12.8–23.8)	18.7 (13.1–25.9)	18.1 (13.0–24.3)	18.1 (13.3–23.1)
	**Men** b	25.9 (18.2–31.6)	26.2 (20.3–34.2)	25.1 (18.9–32.7)	24.8 (21.1–29..0)
**TEE** **(kJ.day^−1^)**	**Women** a	9589 (6803–12664)	9925 (6989–13775)	9646 (6898–12920)	9638 (7081–12287)
	**Men** b	13788 (9668–16827)	13961 (10786–18221)	13353 (10080–17378)	13211 (11234–15431)

a N = 37;

b N = 22;

c N = 59.

### 3.1 Independent Bayesian model

Independent Bayesian modelling of TEE gives results that correlate highly with those from normalized non‐Bayesian methods (overall *r*^2^ = 0.96). This is expected as they are conditional on the data. However, an informative comparison is obtained from a Bland‐Altman plot.^25^ These results indicate that, overall, there is little difference between the two methods. However, for some individuals the discrepancy between normalized non‐Bayesian and independent Bayesian methods is not insignificant. The limits of agreement between the normalized non‐Bayesian and Bayesian methods are much wider for men (−1296 to +1367 kJ·day^−1^) than for women (−750 to +660 kJ·day^−1^).

### 3.2 Hierarchical Bayesian model

When a hierarchical Bayesian analysis is compared with the normalized non‐Bayesian results, there is a marked difference in behaviour between the women and the men. For the men there is an appreciable compression of the distribution obtained from the hierarchical model compared with standard methods (range of TEE is 11234–15431 kJ·day^−1^ compared with 10786–18221 kJ·day^−1^). This is understandable because extremes are drawn into the middle of the population distribution (under the assumption of exchangeability). Although an analogous compression is seen for the women, it is not of such magnitude (Table 2). This compression for men is very apparent in the Bland‐Altman comparison of the two methods, where a pronounced slope is observed on the plot, due to subjects with a low TEE tending towards negative difference and those with a high TEE tending towards a highly positive difference. The summary statistics for the comparison suggest a median difference of −287 kJ·day^−1^ for women and of −750 kJ·day^−1^ for men. Again, the limits of agreement are narrower for women than for men (a range of −92 to +1488 kJ·day^−1^ for women compared with −448 to +2790 kJ·day^−1^ for men). Since TEE is a derived variable in the Bayesian model, no estimates of a hyperparameter are obtained directly. However, in this work, we have chosen to assume a simple scaling factor between $R_{{\mathrm{CO}}_{2}}$ and TEE, and therefore the posterior hyperparameter distributions are obtained indirectly. For the women it is defined by a median of 9638 kJ·day^−1^ with a range from 7081–12287 kJ·day^−1^, while for the men the corresponding values are 13211 kJ·day^−1^ and 11234–15431 kJ·day^−1^, respectively.

## 4 DISCUSSION

In this study we used a Bayesian approach for the estimation of human total energy expenditure (TEE) using doubly labelled water data obtained from 59 participants after incorporating prior information of the space ratio parameter.

In the analysis of methods used to derive TEE from the isotope data, we have shown that $R_{{\mathrm{CO}}_{2}}$ is a linear function of the differences in the isotope effluxes, regardless of the model used. Furthermore, since TEE is taken as proportional to $R_{{\mathrm{CO}}_{2}}$ (i.e. the ratio of macronutrients oxidized is taken to be the same for all subjects), a similar linear relationship must also hold for energy expenditure. Therefore, we write:

(15) $$\mathrm{TEE}=\lambda \left(k_{O}N_{O}-k_{H}N_{H}\right)+\mu =\lambda \left(k_{O}-Sk_{H}\right)N_{O}+\mu$$

when the natural spaces are used. Similarly, normalization of the spaces leads to a relationship:

(16) $$\mathrm{TEE}\prime =\lambda \left(k_{O}-S\prime k_{H}\right){N\prime }_{O}+\mu$$

where the prime denotes the normalization process, which may be summarized by:

(17) $$N\prime_{O}=\frac{1}{2}\left(\frac{S}{S\prime }+1\right)N_{O}$$

Therefore, we expect normalization to change the estimated TEE according to:

(18) $$\mathrm{TE}E\prime -\mathrm{TEE}=\lambda \left(\frac{S}{S\prime }+1\right)\left[\left(\frac{k_{O}+S\prime k_{H}}{2}\right)N_{O}\right]$$

In this expression, the term in square brackets can be regarded as an approximation to the average of the two isotope fluxes, which will be roughly invariant in any population.

On this basis, it might be expected that the application of a Bayesian analysis would produce estimates of TEE midway between those obtained from the natural and normalized methods. However, it must be borne in mind that the usual method of analyzing the disappearance curves uses logarithmic transformation followed by linear least‐squares methods, whereas the formulation that we have used for the Bayesian analysis fits the curves in their exponential form. The question of whether logarithmic transformation is appropriate has been discussed previously, and it has been noted that the correct choice of data pre‐treatment depends upon the error structure of the data,^26^ which is determined by the balance between biological variation and analytical performance. Since the Bayesian approach generates posterior distributions for the fitted data points it is indeed richer in information than the least‐squares method. This is illustrated in Figure 6. This diagram, drawn from ^18^O data for subject 3, shows the measured and predicted ^18^O enrichments (on the *δ*‐scale) for each of the fourteen *post‐dose* timepoints. For ease of comparison, each graph is drawn to the same x‐scale, and centred on the predicted *δ*‐value from the exponential fit. From this figure, it is apparent that, at least for these data, the width of the posterior distribution for the modelled points does not change appreciably with the ^18^O enrichment, apart from in the very early stages of the timecourse, when the ^18^O enrichment is changing rapidly with time, and so errors in the latter are most significant. Under the conditions of constant (non‐proportionate) error in *δ* it is incorrect to use the logarithmic transform.

A second noteworthy point illustrated in Figure 6 is that the mean values of the Bayesian posteriors generally do indeed lie closer to the experimental datapoints than the predictions from the least‐squares estimates. Quantitatively, the root‐mean‐square deviation for the logarithmic fit is 4.00 ‰, that for the exponential fit 3.92 ‰, and 3.89 ‰ for the Bayesian modelling. However, these figures are dominated by the outlying point on day 11; in our opinion this point should have been omitted in the analysis, but we have retained it for consistency with the previous work. Excluding day 11, the rms deviations become 2.9 ‰, 2.6 ‰ and 2.2‰ for the logarithmic, untransformed and Bayesian methods, respectively.

Having demonstrated the utility of the Bayesian method for analysis of DLW data, we chose to examine its performance in a medium‐sized dataset comprising 59 adults (37 women, 22 men). Initially, each subject was modelled individually (independent Bayesian model). In view of the caveat imposed by the anomalous behaviour of the basal ^18^O discussed in the small dataset we first compared the posterior means obtained with the experimental data. In this larger dataset, there was no evidence of non‐ideal behaviour, the root‐mean‐squared residuals between experimental and fitted means being 0.60‰ for ^2^H and 0.20‰ for ^18^O, with limits of agreement from −1.02 to 1.03 ‰ and from −0.41 to 0.39 ‰, respectively. This reassuring result confirms our view that the earlier data^9^ suffered from analytical non‐linearity in the ^18^O data.

A Bland‐Altman analysis of the TEE data showed that there was little overall difference between the normalized non‐Bayesian and independent Bayesian methods, although the limits of agreement were somewhat larger. With these data the limits of agreement for the women (from −750 to +660 kJ·day^−1^) are narrower than those for the men (from −1296 to 1369 kJ·day^−1^); which is probably an artefact due to the relatively small number of subjects, although it does indicate that on an individual basis the difference between the Bayesian and non‐Bayesian result can be far from trivial.

When a Bayesian hierarchical method was used, even when split across men and women, the spread of space ratios was further reduced. In particular, the lower bound is pushed upwards (Figure 3). Under the hierarchical model the range of TEE is also compressed, again with very small overall difference for the women, but now somewhat more for the men. Whilst in both cases there is a significant slope on the Bland‐Altman plot, the slope is much steeper for the men (0.55 compared with 0.17 for the women) indicating that the hierarchical Bayesian method compresses the TEE data considerably more for this group of men than for the women.

Although, for the purposes of assessing Bayesian analysis as a tool for DLW analysis in general, the amount of prior knowledge incorporated into the Bayesian analysis was small, it is reasonable that a higher degree of prior information could be supplied. For example, other anthropometric parameters such as height could be included, and prior assessment of the body composition made using prediction equations such as those derived by Deurenberg and co‐workers or Jackson et al.^27^, ^28^ Studies reported in the research literature on water requirement and turnover are under‐represented compared with studies of other nutrients. Compared with the model presented here, much tighter limits are known to exist and progress is being made in developing prediction equations.^29^ It is even possible to make use of non‐physiological properties of stable isotopes to improve the model. Section S3 (supporting information) illustrates how the meteoric water line can assist in deriving priors for the basal isotopic enrichments. The degree to which informative‐rich priors should be incorporated into the model will depend upon the research question, the homogeneity of the population under investigation, and ultimately on the confidence that the investigator has in his/her prior convictions. In this study, we have used the Bayesian method to address a relatively long‐standing controversy regarding handling of dilution spaces in the DLW method while imposing minimal further restraints upon the determination of TEE. It is an advantage of Bayesian methods that the analysis can be informed as much or as little as is deemed appropriate for dealing with the particular experimental circumstances.

In principle, a Bayesian method is a stochastic approach where the parameter of interest has an assumed probability distribution (prior) which is updated by the observed dataset to generate the parameter's posterior distribution. If in an extreme case where the measurement error is zero and the underlying mechanistic model is true, the prior information about the parameter will be considered of zero weight. As such, the model will fit perfectly into the dataset and thus the estimation of the parameter (for example TEE) will be an error‐free value resulting in the same estimation as if a least‐squares method is used. In any other case, the prior information used in the Bayesian method will play a role in the estimation of the posterior distribution and if the prior information is valid this will increase the estimation accuracy. In effect, when the laboratory precision is limited, the use of Bayesian methods could improve the estimations of TEE to that of a laboratory in which a high level of instrument precision is observed. Where multi‐subject datasets are available, a hierarchical model can be further applied that results in an even more precise estimation of TEE.

## 5 CONCLUSIONS

Bayesian analysis is an appealing approach to estimate population and individual total energy expenditure with the doubly labelled water method. The method offers a valuable approach to deal with outliers and missing data and gives a smaller unbiased estimate on the population dispersion, particularly if a hierarchical model is used.

## Supporting information




**Data S1.** Transformation between kinetic and physiological parameters.
**Data S2**. Derivation of suggested prior distributions for the basal enrichment
**Data S3.** Parameter estimation

## ENDNOTE

*
In the absence of isotope fractionation, $R=\frac{k_{O}N_{O}-k_{H}N_{H}}{2}$. There has been much discussion on the corrections applicable to this basic equation to account for differential loss of isotope; however, nearly all lead to an equation of the form of Equation (4) with the coefficients *α*_1_ and *α*_2_ dependent on the model used. For example, using literature values for fractionation factors, the model of Schoeller et al. gives *α*_1_ = 0.4589, *α*_2_ = 0, while Coward's proposal yields *α*_1_ = 0.4689, α_2_ = −0.6495. Similar considerations apply to the other parameters. In the remainder of this work we will adopt Coward's model, for which *β*_1_ = 0.9676, *β*_2_ = 1.5544, *γ*_1_ = 0.01187 kg.mol^−1^ and *γ*_2_ = 0.01222 kg.mol^−1^.
