# Supplementary material for: Application of Bayesian analysis to the doubly labelled water method for total energy expenditure in humans
Source: Rapid Commun Mass Spectrom. 2017 Nov 23;32(1):23–32. doi: 10.1002/rcm.8013 (PMC5765481; doi:10.1002/rcm.8013)
Supplement: Supplementary file 1 — Data S1. Transformation between kinetic and physiological parameters. Data S2. Derivation of suggested prior distributions for the basal enrichment Data S3. Parameter estimation [file RCM-32-23-s001.docx]

### Supplementary Information 1– transformation between kinetic and physiological parameters.

The isotope (tracer) disappearance from the human body is not exactly equivalent to tracer kinetics because of isotopic fractionation. In the early years of the application of the DLW technique, there was much debate as to how this should be handled, and this was eventually resolved in terms of two models, one due to [1], and one to [3]. Both begin with the same fundamental reasoning, but differ in their implementation. However, general equations can be derived, for which substitution of the appropriate constant coefficients will give either Schoeller’s or Coward’s solution, as will now be derived. Water can be assumed to leave the body by three different routes: lost as liquid, $R_{L};$ lost transcutaneously, $R_{T};$ and lost in breath, $R_{B}$. The latter two routes are fractionated. In addition oxygen from water is lost via incorporation into breath with an efflux of $R_{{CO}_{2}}$. Taking fractionation into account leads to a pair of equations describing the efflux of ^18^O and ^2^H ($R_{O}$ and $R_{H}$) respectively as

$$\begin{matrix} R_{O}=2f_{3}R_{{CO}_{2}}+R_{L}+f_{2}\left( R_{T}+R_{B} \right) \\ R_{H}=R_{L}+f_{1}\left( R_{T}+R_{B} \right) \end{matrix}$$

where the coefficients are fractionation factors $f_{1}$, $f_{2}, and f_{3}$and defined in Prentice [2], as *f_1_* being the ^2^H_2_O vapour/liquid fraction, *f_2_* being the H_2_^18^O vapour/liquid fraction and *f_3_* the C^18^O_2_/H_2_^18^O ratio. Since the total water loss is given by

$$R_{W}=R_{L}+R_{T}+R_{B}$$

$$R_{O}=2f_{3}R_{{CO}_{2}}+R_{W}-\left( 1-f_{2} \right)\left( R_{T}+R_{B} \right)$$

$$R_{H}=R_{W}-\left( 1-f_{1} \right)\left( R_{T}+R_{B} \right)$$

These can be rewritten explicitly as simultaneous equations in $R_{{CO}_{2}}$ and $R_{W}$ yielding

$$\begin{matrix} R_{{CO}_{2}}=\frac{R_{O}-R_{H}-\left( f_{2}-f_{1} \right)\left( R_{T}+R_{B} \right)}{2f_{3}} \\ R_{W}=R_{H}+\left( 1-f_{1} \right)\left( R_{T}+R_{B} \right) \end{matrix}$$

Schoeller’s and Coward’s analyses now diverge as they make different assumptions for $R_{T}$ and $R_{B}.$ Both assume that there is a constant rate between exhaled CO_2_ and water, and therefore $R_{B}\propto R_{{CO}_{2}}$, albeit with different constants of proportionality. Schoeller assumes that the average concentration of CO_2_ in breath is 3.5%, and that breath is 95% saturated with water at 36°C, and therefore that exhaled air at atmospheric pressure comprises 5.56% water vapour, which gives

$$R_{B}(Schoeller)=\frac{0.056}{0.035}R_{{CO}_{2}}=1.59R_{{CO}_{2}}$$

In contrast, however, Coward used a measured value of 0.83g of liquid water per litre of exhaled CO_2,_ which, see footnote^[[1]](#footnote-1)^, is equivalent to,

$$R_{B}(Coward)=1.17R_{{CO}_{2}}$$

For $R_{T}$ Schoeller also assumes a proportional relationship with $R_{{CO}_{2}}$.with a ratio of 0.5 for adults. However, Coward suggests that this term should be taken as a constant, which he assumes to be 500g/day or 27.7moles/day. Using these various assumptions and equations for CO_2_ production and water efflux, Schoeller’s and Coward’s models can be expressed as

$\left. \begin{matrix} R_{{CO}_{2}}=\frac{R_{O}-R_{H}}{2f_{3}+2.1\left( f_{2}-f_{1} \right)}=0.4589\left( R_{O}-R_{H} \right) \\ R_{W}=\frac{\left[ 2f_{3}-2.1\left( 1-f_{2} \right) \right]R_{H}+2.1\left( 1-f_{1} \right)R_{O}}{2f_{3}-2.1\left( f_{2}-f_{1} \right)}=1.0437R_{H}+0.0629R_{O} \end{matrix} \right\}$ Schoeller

$\left. \begin{matrix} R_{{CO}_{2}}=\frac{R_{O}-R_{H}-27.7\left( f_{2}-f_{1} \right)}{2f_{3}+1.17\left( f_{2}-f_{1} \right)}=0.4689\left( R_{O}-R_{H} \right)-0.6495 \\ R_{W}=R_{H}+\left( 1-f_{1} \right)\left( 27.7+1.17\frac{R_{O}-R_{H}+27.7\left( f_{2}-f_{1} \right)}{2f_{3}-1.17\left( f_{2}-f_{1} \right)} \right)=0.9658R_{H}+0.03425R_{O}+1.6817 \end{matrix} \right\}$ Coward

where the accepted values $f_{1}=0.941$, $f_{2}=0.991$, $f_{3}=1.037$ have been inserted.

In general terms, then we can write model-independent equations

$$\left. \begin{matrix} R_{{CO}_{2}}=\alpha_{1}\left( R_{O}-R_{H} \right)+\alpha_{2}=\alpha_{1}\left( k_{O}N_{O}-k_{H}N_{H} \right)+\alpha_{2} \\ R_{W}=\beta_{1}R_{H}+\beta_{2}R_{O}+\beta_{3}=\beta_{1}k_{H}N_{H}+\left( 1-\beta_{1} \right)k_{O}N_{O}+\beta_{2} \end{matrix} \right\}$$

and choose the coefficients $\alpha_{1}$, $\alpha_{2}$, $\beta_{1}$, and $\beta_{2}$ to suit the model under discussion

The space ratio is defined, as is customary, by

$$S=\frac{N_{H}}{N_{O}}$$

However there is no information from the DLW experiment to allow the definitive calculation of the body water pool size, $N$, and hence make estimates of body composition. Usually if $N$ is derived from hydrogen data it is assumed that $N_{H}=1.04N$, and if from oxygen that $N_{O}=1.01N$. Taken together this amounts to fixing the space ratio at 1.03, in contrast to our requirement that this parameter be experimentally determined. However, there is no *a priori* reason for preferring one of these estimates to the other, and so they should be somehow combined for our estimation of $N$. It is proposed that the weighted average be adopted; therefore

$$N=\frac{1}{2}\left( \frac{N_{H}}{1.04}+\frac{N_{O}}{1.01} \right)$$

is suggested^[[2]](#footnote-2)^. This is combined with a hydration factor of 73% for lean tissue [3] and a two-compartment model of body composition to obtain an estimate of the fraction of body fat as

$$F=1-\frac{0.01802}{2\times0.73\times W}\left( \frac{N_{H}}{1.04}+\frac{N_{O}}{1.01} \right)$$

$W$ being the weight of the subject in kg, and 0.01802 kg.mole^-1^ the relative molar mass of naturally abundant water. This equation can also be written in a generalised form as

$F=1-\frac{\gamma_{1}}{W}N_{H}-\frac{\gamma_{2}}{W}N_{O}$.

Just as we can define the physiologically meaningful parameters in terms of the isotope kinetics

$$\begin{matrix} R_{{CO}_{2}}=\alpha_{1}\left( k_{O}N_{O}-k_{H}N_{H} \right)+\alpha_{2} \\ R_{W}=\beta_{1}k_{H}N_{H}+\left( {1-\beta}_{1} \right)k_{O}N_{O}+\beta_{2} \\ F=1-\frac{\gamma_{1}}{W}N_{H}-\frac{\gamma_{2}}{W}N_{O} \\ S=\frac{N_{H}}{N_{O}} \end{matrix}$$

the equations may be posed as the inverse where the isotope kinetics are defined by the physiological entities

$$\begin{matrix} N_{H}=\frac{WS\left( 1-F \right)}{\gamma_{1}S+\gamma_{2}} \\ N_{O}=\frac{W\left( 1-F \right)}{\gamma_{1}S+\gamma_{2}} \\ k_{H}=\frac{\left( \gamma_{1}S+\gamma_{2} \right)}{W\left( 1-F \right)}\frac{\left[ \alpha_{1}\left( R_{w}-\beta_{2} \right)-\left( 1-\beta_{1} \right)\left( R_{{CO}_{2}}-\alpha_{2} \right) \right]}{\alpha_{1}S} \\ k_{O}=\frac{\left( \gamma_{1}S+\gamma_{2} \right)}{W\left( 1-F \right)}\frac{\left[ \alpha_{1}\left( R_{w}-\beta_{2} \right)+\beta_{1}\left( R_{CO2}-\alpha_{2} \right) \right]}{\alpha_{1}} \end{matrix}$$

### Supplementary Information 2 – Parameter estimation

We estimated the model parameters through Bayesian inference [5] with and without a hierarchical approach [6]. According to Bayes Theorem,

Where, in the present study, *y* is the dataset of the oxygen/hydrogen isotope enrichment measurements, θ represent the unknown individual parameters comprising $R_{{CO}_{2}}$, *S*, *R_W_* and *F*. The prior knowledge on these parameters is represented by prior distribution *p(θ)* which is updated using the likelihood *p(y|θ)* to yield parameters’ posterior distributions

*p(θ|y)* =

which performs as a scaling factor.

Within the Bayesian hierarchical framework, the prior *p(θ)*  can be further decomposed into conditional distributions

.

The marginal prior distribution for *θ* is then

where *φ_k_* can be referred to as the hyperparameters of level *k* and the conditional prior distributions *p*(*φ_k-1_* |* φ_k_*) expressing structural judgements. In the present analysis, *k* equals to 2 as we assumed that the subject-level parameters were exchangeable and were samples drawn from a higher-level distribution with population-level parameter mean and variance hyperparameters (*φ_2_*) to which we assigned hyperpriors. The hyperparameters were estimated in parallel with the subject-level parameters. An important characteristic of hierarchical models is that each parameter, referring to a specific unit (subject), borrows strength from the corresponding parameters of other units (subjects) with similar characteristics. In other words, a shrinkage effects towards the population mean emerges with the use of hierarchical models. Generally, hierarchical models are more flexible than typical non-hierarchical (fixed effects) models because the more complicated structure is accommodated in the model. Bayesian inference Using Gibbs Sampling (here WinBUGS) [7] was used for Bayesian inference employing Markov chain Monte Carlo (MCMC) and Metropolis-Hasting algorithm to approximate the posterior distribution *p(θ |y)*.

### Supplementary Information 3-Derivation of suggested prior distributions for the basal enrichment

Assuming that body water is derived from a source which has not undergone excessive evaporation, it is expected that the isotopic enrichments of ^2^H and ^18^O will display a linear correlation [4]. The slope of this line (the meteoric water line) is such that there is an eightfold increment in δ ^2^H for unit increment in δ ^18^O. This equation can be used to impart prior knowledge to the measured basal isotope enrichments in hierarchical or non- hierarchical DLW experiments as follows:

If the relationship expressed by the meteoric water line was exact,

$$\Delta_{bH}=8\Delta_{bO}+c$$

where the upper case deltas indicate the priors. In meterology, the value of the constant $c$ (the ‘deuterium excess’) is taken as 10‰ for the global meteoric water line; however, it is known to deviate from this value in many instances. For our purposes, this is unfortunate since this precludes us from predicting the relationship between the basal enrichments for a single subject.

However, if we are dealing with several subjects simultaneously (whether hierarchically or not) we can use a form of linear regression to obtain a degree of prior information on the likely relationship. To do this, we first note that any linear regression must pass through the centre of gravity of the data points, and therefore the value of the deuterium excess for the population under consideration must be

$$c=\frac{\sum_{i=1}^{n} \delta_{bH}[i]-8\sum_{i=1}^{n} \delta_{bO}[i]}{n}=\bar{\delta_{bH}}-8\bar{\delta_{bO}}$$

where the sum has been taken over all $n$ subjects. Consider now the $k$th subject, for which the basal enrichments are measured as $\delta_{Ok}$, $\delta_{Hk}$. Our prior assumption is that these values should lie on the meteoric water line, at the point where it is intersected by a line projected from the observed data with direction cosines ($\sigma_{O,}$ $\sigma_{H}).$ Therefore,

$$\begin{matrix} \Delta_{bO}[k]=\delta_{bO}[k]-\lambda[k]\sigma_{O} \\ \Delta_{bH}[k]=\delta_{bH}[k]-\lambda[k]\sigma_{H} \end{matrix}$$

However from the earlier analysis

$$\Delta_{bH}[k]-\bar{\delta_{bH}}=8\left( \Delta_{bO}[k]-\bar{\delta_{bO}} \right)$$

and therefore,

$$\lambda[k]=\frac{\left( \delta_{bH}[k]-\bar{\delta_{bH}} \right)-8\left( \delta_{bO}[k]-\bar{\delta_{bO}} \right)}{\left( \sigma_{H}-8\sigma_{O} \right)}$$

Our prior distributions are therefore

$$\begin{matrix} \Delta_{bH}\left[ k \right]\sim dnorm\left( \delta_{bH}\left[ k \right]-\lambda\left[ k \right]\sigma_{H}, \frac{1}{\sigma_{H}^{2}} \right) \\ \Delta_{bO}\left[ k \right]\sim dnorm\left( \delta_{bO}\left[ k \right]-\lambda\left[ k \right]\sigma_{O}, \frac{1}{\sigma_{O}^{2}} \right) \end{matrix}$$

**References**

1. Schoeller DA, Ravussin E, Schutz Y, Acheson KJ, Baertschi P, Jequier E. Energy Expenditure by Doubly Labelled Water: Validation in Humans and Proposed Calculation. *Am J Physiol* 1986, 250:R823-830.

2. Prentice A (Ed.). The Doubly-labelled water method for measuring energy expenditure - Technical recommendations for use in humans. Vienna: International Atomic Energy Agency; 1990.

3. Pace N, Rathbun EN. Studies on Body Composition, III. The Body ater and Chemically Combined Nitrogen Content in Relation to Fat Content. *J Biol Chem* 1945, 158:685-691.

4. Craig H: Standard for Reporting Concentrations of Deuterium and Oxygen-18 in Natural Waters *Science* 1961, 133:1833-1834.

5. Gelman A. *Bayesian Data Analysis.* 2nd edn. Boca Raton, Fla.: Chapman & Hall/CRC; 2004.

6. Ntzoufras I: *Bayesian Modeling Using WinBUGS.* Hoboken, N.J.: Wiley; 2009.

7. Lunn DJ, Thomas A, Best N, Spiegelhalter D. WinBUGS - A Bayesian modelling framework: Concepts, structure, and extensibility. *Stat Comput* 2000, 10:325-337.

1. The conversion is achieved by dividing the weight of liquid water by 18.02 to obtain moles, multiplying by 22.4 to express as litres of water vapour at 273K, and finally multiplying by (36+273)/273 to find the volume of water vapour at body temperature. Therefore, breath containing 1g liquid water per litre of CO_2_ has a water vapour to CO_2_ ratio of $\left( 22.4\times309 \right)/{\left( 18.02\times273 \right)=1.407}.$ It is noteworthy that in Coward’s 1988 paper (3) the temperature correction is applied inversely in error, and that the experimental value of 0.83 mg liquid water per litre of CO_2_ corresponds to a more plausible 70% of the saturated vapour pressure of water at 36°C, rather than the 55% reported in that article. [↑](#footnote-ref-1)
2. It is important to appreciate the difference between the use of this weighting to obtain an estimate of body composition, and its use in Schoeller’s space ratio normalization for *TEE*. [↑](#footnote-ref-2)
